# Bimanual motor skill learning with robotics in chronic stroke: comparison between minimally impaired and moderately impaired patients, and healthy individuals

**DOI:** 10.1186/s12984-022-01009-3

**Published:** 2022-03-17

**Authors:** Eloïse Gerardin, Damien Bontemps, Nicolas-Thomas Babuin, Benoît Herman, Adrien Denis, Benoît Bihin, Maxime Regnier, Maria Leeuwerck, Thierry Deltombe, Audrey Riga, Yves Vandermeeren

**Affiliations:** 1 Neurology Department, Stroke Unit, UCLouvain, CHU UCL Namur (Godinne), Yvoir, Belgium; 2 Louvain Bionics, UCLouvain, Louvain-la-Neuve, Belgium; 3 Institute of NeuroScience (IoNS), NEUR Division, UCLouvain, Brussels, Belgium; 4 Department of Physical Medicine and Rehabilitation, UCLouvain, CHU UCL Namur (Godinne), Yvoir, Belgium; 5 Faculty of Motor Sciences, UCLouvain, Louvain-La-Neuve, Belgium; 6 Institute of Mechanics, Materials and Civil Engineering, UCLouvain, Louvain-la-Neuve, Belgium; 7 Scientific Support Unit (USS), UCLouvain, CHU UCL Namur (Godinne), Yvoir, Belgium

**Keywords:** Motor learning, Bimanual, Bimanual coordination, Robotics, Stroke, Neurorehabilitation

## Abstract

**Background:**

Most activities of daily life (ADL) require cooperative bimanual movements. A unilateral stroke may severely impair bimanual ADL. How patients with stroke (re)learn to coordinate their upper limbs (ULs) is largely unknown. The objectives are to determine whether patients with chronic supratentorial stroke could achieve bimanual motor skill learning (bim-MSkL) and to compare bim-MSkL between patients and healthy individuals (HIs).

**Methods:**

Twenty-four patients and ten HIs trained over 3 consecutive days on an asymmetrical bimanual coordination task (CIRCUIT) implemented as a serious game in the REAplan® robot. With a common cursor controlled by coordinated movements of the ULs through robotic handles, they performed as many laps as possible (speed constraint) on the CIRCUIT while keeping the cursor within the track (accuracy constraint). The primary outcome was a bimanual speed/accuracy trade-off (biSAT), we used a bimanual coordination factor (biCO) and bimanual forces (biFOP) for the secondary outcomes. Several clinical scales were used to evaluate motor and cognitive functions.

**Results:**

Overall, the patients showed improvements on biSAT and biCO. Based on biSAT progression, the HI achieved a larger bim-MSkL than the patients with mild to moderate impairment (Fugl-Meyer Assessment Upper Extremity (FMA-UE): 28–55, n = 15) but not significantly different from those with minimal motor impairment (FMA-UE: 66, n = 9). There was a significant positive correlation between biSAT evolution and the FMA-UE and Stroke Impact Scale.

**Conclusions:**

Both HI and patients with chronic stroke training on a robotic device achieved bim-MSkL, although the more impaired patients were less efficient. Bim-MSkL with REAplan® may be interesting for neurorehabilitation after stroke.

*Trial registration*: ClinicalTrial.gov identifier: NCT03974750. Registered 05 June 2019. https://clinicaltrials.gov/ct2/show/NCT03974750?cond=NCT03974750&draw=2&rank=1

**Supplementary Information:**

The online version contains supplementary material available at 10.1186/s12984-022-01009-3.

## Background

Stroke is a major cause of long-term disability worldwide [1, 2], and one of the most frequent impairments is hemiparesis, which is characterized by weakness, lack of control, increased muscle tone on the contralesional upper limb (UL) and lower limb or hemibody, and deteriorating independence in activities of daily life (ADL), especially walking, dressing or eating [3]. Critically, other impairments (e.g., somatosensory, visual, and cognitive), whether isolated or combined, also significantly deteriorate ADL. Most ADL require skilled bimanual coordination that can be impaired by a stroke, thus leading to a loss of independence that may in turn lead to a 50% reduction in quality of life [4]. Despite rehabilitative care provided during the acute phase of stroke, 30% of patients still suffer from participation restrictions after four years [5]. It has been suggested that neurorehabilitation should not focus exclusively on impairments of the paretic arm or hand and should instead consider more bimanual actions and activities [6–8]. After a unilateral stroke, impairments of the contralesional UL can deteriorate bimanual actions [9], thus supporting the importance of training both ULs to achieve better functional recovery in (bimanual) ADL [8, 10]. Interestingly, during bilateral cooperative movements (e.g., opening a bottle), neural coupling from the ipsilesional to the contralesional (impaired) UL is preserved in most patients with stroke, suggesting the relevance of bilateral training that supports cooperative hand movements for ADL [11]. During bimanual training, various tasks can be utilized to promote intensive and repetitive coordinated movement of the ULs. A classification of bimanual tasks has been proposed for different bimanual actions. Grossly, two types of tasks can be distinguished: those with symmetrical movements that engage homologous muscles (e.g., picking up a box simultaneously with both hands) and those with asymmetrical movements that engage nonhomologous muscles nonsimultaneously (e.g., cutting a piece of steak). Similarly, two types of task goals can be distinguished: independent goals (e.g., one hand lifting a cup and the other hand lifting a glass simultaneously) versus common goals (e.g., both hands working together to accomplish a common task) [8]. Many bilateral actions, such as arms swinging during bipedal locomotion, seem to depend on “default-mode” neural coupling. However, in most skilled ADL, bimanual actions are accomplished through asymmetrical movements that cooperate to achieve a common goal, e.g., buttoning a skirt or changing the gear while steering a car. Such complex bimanual, cooperative, asymmetrical skills have to be learned.

After a stroke, motor skill learning (MSkL) plays a key role in recovery by compensating for activity limitations and participation restrictions. MSkL is a fundamental ability that allows for the acquisition of unimanual or bimanual skills (i.e., writing, playing the piano) and adaptation of these skills to changing environments. It has been suggested that procedural learning, including MSkL, proceeds over three phases [12]: (i) an early “strategic/cognitive” phase, which presents rapid performance improvement, especially in the dorsolateral prefrontal cortex and posterior parietal cortex (PPC); (ii) a consolidation phase, which involves the stabilization of the learned skill based principally through a corticostriatal loop (striatum and supplementary motor area); and (iii) a retention phase, which is also called the “automatization phase”, during which the performance of the skill is optimized due to the increased activity in the primary motor cortex (M1), the premotor cortex and PPC [13]. Improvement of a skill is linked to practice-dependent training: the more we practice a skill, the better we perform it, with smoother movements and reduced variability [14, 15]. Sensorimotor skill acquisition (or MSkL) represents the ability to select and refine the movements needed to attain a goal in which the sensory stimuli for selecting and correcting our actions are considered and then the skill is executed consistently with both speed and accuracy (i.e., with motor acuity). Once learned, a motor skill can be retained for long periods of time, thus leading to lasting performance improvements, which is the aim of neurorehabilitation [16].

Robotic devices have long been expected to enhance recovery after a brain injury, such as a stroke [17–20], because they offer the possibility of providing intensive task-specific training to regulate task parameters, quantify and monitor improvements, and continuously adapt the task’s difficulty [21, 22]. E.g., Keeling and al. [20] showed that the use of the bimanual robotic tasks for rehabilitation in subacute stroke is feasible and suggested that the use of robotic devices added to standard of care therapy could augment recovery. Moreover, the proprioceptive feedback during active movements, delivered through a robotic therapy improved sensorimotor function in chronic stroke patients [23]. In fact, the proprioceptive training could enhance somatosensory and motor functions and induce cortical reorganization [24]. Interestingly, robotics has the potential to formally implement the principles of motor learning in neurorehabilitation. Cuppone et al. [25] found that somatosensory learning is linked to motor learning because these processes similar features of memory formation. Robotic devices can provide four main training modalities: (i) active mode (where the subject fully performs the task), (ii) active-assisted mode (where the robot provides assistance either at a fixed rate or “as needed”), (iii) passive mode (where the robot fully performs the task), and (iv) resistive mode (where the robot perturbs the subject’s attempts); these modalities allow for valuable interactions with patients [26]. In a meta-analysis, Kwakkel et al. showed significant improvement in UL impairment (but not in ADL) with robot-assisted training (RAT) [27]. However, a recent Cochrane review showed that RAT enhances both UL impairments and ADL in stroke survivors [18], and another team showed that RAT improved motor coordination compared to unilateral training in patients with stroke with severe impairments [28].

More recently, we used a custom system with computer mice and showed that patients with stroke were able to learn, retain and generalize a complex bimanual skill after a single session of real and sham transcranial direct current stimulation (tDCS) [29]. Determining whether patients with stroke could achieve bimanual MSkL (bim-MSkL) and identifying the underlying mechanisms and extent of learning are crucial for the development of efficient neurorehabilitation approaches targeting independence in (bimanual) ADL.

To explore how patients (re)learn to coordinate their hands after a stroke, we developed a complex asymmetrical bimanual coordination task (CIRCUIT) that was implemented as a serious game in the bimanual version of the REAplan® robot (AXINESIS, Wavre, Belgium). With a common cursor controlled by coordinated movements of the ULs interacting with robotic handles, one hand exclusively controlled lateral displacements of the common cursor while the other hand exclusively controlled the sagittal displacements. It has been suggested that stroke recovery studies should include quantitative measures, such as speed, accuracy, path length metrics and smoothness of movement [26]. By analyzing kinematic parameters and providing such real-time quantitative measures of movement, REAplan® can be used for training UL movements [30, 31]. Our hypotheses were that (i) patients in the chronic phase of stroke would show improvements in a new complex bimanual coordination skill and be able to retain and generalize this skill, i.e., they would be able to achieve complex bim-MSkL and/or improve on other bimanual or unimanual performances; (ii) patients would show similar improvements in bim-MSkL as healthy individuals; and (iii) poorer baseline clinical scales in patients would correlate with poorer bim-MSkL indices.

## Methods

### Participants

Twenty-four patients in the chronic phase of stroke (> 6 months, Table 1) and ten healthy individuals (HIs) were recruited between July and November 2019 at CHU UCL Namur (Godinne site, Belgium) after providing written informed consent. All procedures were approved by the local ethics committee and complied with the Declaration of Helsinki. For the patients, the inclusion criteria were as follows: (1) aged 18–85 years old, (2) history of supratentorial stroke demonstrated by cerebral imaging, and (3) ability to complete three consecutive days of training with a robotic device. The exclusion criteria were as follows: (1) major difficulty understanding or executing commands, (2) drug or alcohol abuse, (3) severe aphasia or cognitive deficits that interfered with the study, (4) inability to voluntarily move the affected arm (i.e., complete paralysis), and (5) multiple strokes, dementia, or psychiatric conditions. For HIs, the inclusion/exclusion criteria were as follows: (1) 18–85 years old, (2) no neurological condition, (3) no drug or alcohol abuse, and (4) no psychiatric conditions. None of the patients nor HI were familiar with the REAplan.

**Table 1:** Demographic characteristics of the patients with stroke

		HIs	All patients	Group 1	Group 2
		N = 10	N = 24	N = 9	N = 15
FMA-UE	Median (min; max)	66 (66; 66)	64 (28; 66)	66 (66; 66)	59 (28; 65)
Female	N (%)	6 (60)	11 (46)	5 (55)	6 (42)
Age	mean (SD)	64 (11)	61 (11)	66 (9)	57 (12)
Time since stroke	6–12 months		1 (4)	0 (0)	1 (7)
	12–36 months		9 (38)	3 (33)	6 (42)
	> 36 months		14 (58)	6 (66)	8 (56)
Localisation	Cortical		13 (54)	5 (55)	8 (56)
	Subcortical		10 (42)	4 (44)	6 (42)
MoCA	Median (min; max)	(25; 30) *	27 (8; 30)	27 (23; 30)	27 (8; 29)
ABILHAND	Median (min; max)		2.8 (− 0.9; 6.0)	4.4 (1.2; 6)	1.3 (− 0.9; 4)
SIS	Median (min; max)		74 (42; 99)	89 (57; 99)	68 (42; 93)

*FMA-UE* Fugl-Meyer upper extremity, *SD* standard deviation, *MoCA* Montreal Cognitive Assessment, *ABILHAND* bimanual activity limitation of ADL (questionnaire), *SIS* stroke impact scale

*Normative value for healthy individuals (HIs)

### Clinical assessment

Several clinical scales and tests were used to evaluate motor and cognitive functions based on the International Classification of Functioning, Disability and Health (ICF) [32]. We evaluated motor impairment of the UL with the Fugl-Meyer Upper Extremity (FMA-UE) [33], hand dexterity with the Box and Blocks Test (BBT) [34], grip force (GF) with a Jamar dynamometer [35], and cognitive impairment with the Montreal Cognitive Assessment (MoCA) [36]. Bimanual activity limitation of ADL was evaluated with the ABILHAND questionnaire [37], and participation restrictions were measured with the Stroke Impact Scale (SIS) [38].

### Study design

**Fig. 1:**
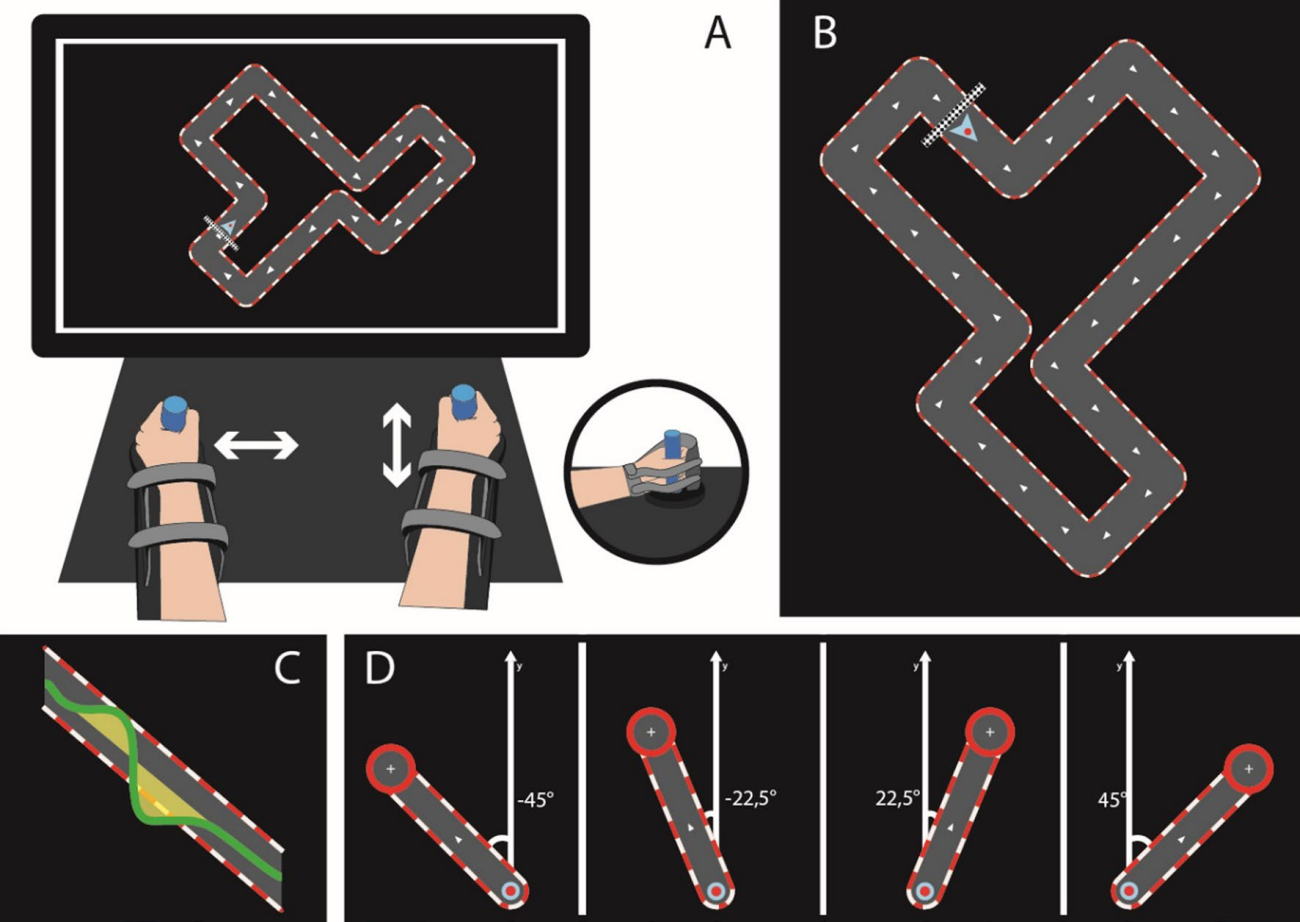
Bimanual tasks on the REAplan®. **A** General setup of the bimanual version of the REAplan® robot. Note that each hand slid exclusively along one axis and thus controlled a different direction of the common cursor (small arrowhead) displayed on the REAplan® screen. The forearms rested in gutters and were strapped in, and handles were adapted if needed. **B** Different circuit of identical length and difficulty for the generalization. **C** Cursor displacement with regard to the ideal trajectory defined as the center of the circuit track (surface = error), **D** REACHING task: four eccentric targets designated in a pseudorandomized order (16 trials/target)

Over three consecutive days, the participants were evaluated and trained on a bimanual neurorehabilitation robot. On day 1 (D1), after clinical assessment and a short familiarization period with the REAplan®, the participants performed a bimanual REACHING task. Next, they started the bimanual CIRCUIT task (Fig. 1a), with a baseline evaluation that consisted of three blocks of 1 min (with 30 s rest intervals), followed by 20 training blocks of 1 min (interlaced with rest blocks of 30 s). On day 2 (D2), training was repeated identically. During the last day (D3), after completing the same training again, the participants performed a new bimanual circuit (NC, Fig. 1b) to assess the generalization of the bim-MSkL (3 times: 1 min training/30 s rest), and the BBT and GF were administered as on D1 to assess for transfer on unimanual performance. Bimanual REACHING was also evaluated on D3 after the training and the generalization (NC) evaluation, to assess for generalization within the same robotic environment but with a different task.

The online minimization software QMinim® (http://rct.mui.ac.ir/q/index.php) was used for randomization. It provided the bimanual configuration (i.e., which direction of the common cursor the paretic UL controlled: lateral versus sagittal) to balance between subjects, in which the UL controlled each direction. The patients were thus randomized into two groups, and the randomization criteria were as follows: sex (M/F), age (< 60 / > 60 years), time since stroke (6–12 months/1–3 years/ > 3 years), stroke laterality (dominant/nondominant hemisphere), baseline FMA-UE (< 28/29—42 / > 43), and stroke localization (cortical/subcortical). The HIs were also randomized into two groups with QMin® using the following criteria: sex (M/F), age (< 60/ > 60 years), handedness (left-handed/right-handed), and school level (< 12/ > 12 years).

### Bimanual tasks

The REAplan® (AXINESIS, Wavre, Belgium) is a neurorehabilitation robot with a distal effector that allows for work in two dimensions in the horizontal plane and precisely quantifies movement kinematics and forces through position and force sensors sampled at 80 Hz (Additional file 1). The bimanual version of the REAplan® was used in this study.

The participants practiced several visuomotor tasks on the REAplan® that required complex, coordinated movements with both ULs, i.e., complex bimanual tasks. Coordinated movement of both hands was required to control the displacement of a common cursor. Each hand controlled the cursor’s displacement along a single axis, with either left–right or front-back motions (i.e., lateral X-axis or sagittal Y-axis, Fig. 1a). The movements of each hand were constrained by virtual walls, i.e., forces exerted on the handles by the robot. For example, if the right hand controlled sagittal displacements of the cursor (Y-axis), then horizontal movements were prevented by the virtual walls. The randomization determined which direction (X-axis or Y-axis) was controlled by the paretic UL or the nondominant UL of HIs.

Two serious games (CIRCUIT and REACHING, Fig. 1a/b) were implemented in the bimanual REAplan®. The CIRCUIT is a serious game designed for MSkL that we have previously used in HIs and patients with stroke, including both unimanual or bimanual versions [39, 40]. The bimanual CIRCUIT is a complex asymmetrical bimanual coordination task requiring the learning of a new bimanual control policy [29, 41]. With a common cursor controlled by coordinated movements of the ULs (Additional file 1), the subjects were instructed to perform as many laps as possible on a complex circuit during the 1-min blocks (i.e., the speed constraint: “as fast as possible”) while keeping the cursor within the track (i.e., the accuracy constraint, the cursor had the same width as the track of the CIRCUIT). At the end of each block, a block score (reflecting the bimanual speed/accuracy trade-off (biSAT, see below)) and a high score (i.e., best score thus far) were displayed to motivate the subject.

For the bimanual REACHING task, the subjects were instructed to reach four eccentric targets designated in a pseudorandomized order (16 trials/target). The subjects had to quickly move from the home position to the current target using coordinated bimanual movements and keep the cursor steady on the target for 300 ms before being instructed to actively move back to the home position and wait (100 ms) for the next trial. With respect to the midline (Y-axis), the targets were displayed at ± 45° (symmetrical involvement of each hand, which was identical to the coordination needed for the CIRCUIT, Fig. 1c) or ± 22.5° (asymmetrical hand involvement, Fig. 1d). At the end of the block, a high score was displayed.

### Data analysis

For the analysis of the robotic serious game data (CIRCUIT and REACHING), MATLAB routines (2018b, MathWorks Inc, Natick, MA, United States) were developed. The raw data were resampled in 3-s bins. The following outcomes were computed [29, 41].

1. The primary outcome used to quantify training-induced improvement was a bimanual speed/accuracy trade-off (biSAT) in arbitrary units (a.u.):
  $$1. \,\, biSAT=\frac{speed \, (\frac{cm}{s})}{error \, (c{m}^{2})}.C \, (\mathrm{Where \, C}\hspace{0.17em}=\hspace{0.17em}1\mathrm{ \, cm s})$$
  Speed was the first derivative of the common cursor position. Errors were quantified based on the surface between the ideal path (defined as the center of the track) and the real trajectory of the cursor (Fig. 1c).
  The secondary outcomes were as follows.
2. The bimanual coordination factor (biCO) quantified how well the hands’ movements were coordinated. The ideal biCO corresponds to an equal speed of both hands along the X- and Y-axes:
  $$2. \,\, \mathrm{ biCO}=\frac{min(\left|{V}_{x}\right|,\left|{V}_{y}\right|)}{\sqrt{{\left({V}_{x}\right)}^{2}+{\left({V}_{y}\right)}^{2}}}$$
  where the numerator “$min(\left|{V}_{x}\right|,\left|{V}_{y}\right|)$” represents the minimum value between $\left|{V}_{x}\right|$ (horizontal displacement of hand velocity) and $\left|{V}_{y}\right|$ (vertical displacement of hand velocity) and the denominator ($\sqrt{{\left({V}_{x}\right)}^{2}+{\left({V}_{y}\right)}^{2}})$ corresponds to the velocity of both ULs.
  For the REACHING task, the biCO formula was modified for targets at ± 22.5° angles (see formula as Additional file 1: Fig. S1).
3. The bimanual forces exerted against the virtual walls (biFOP) correspond to the forces exerted in nondesired directions for each hand (i.e., against the virtual walls) in newtons (N):
  $$3. \left|\left|biFOP\right|\right|= \sqrt{{F}_{y}^{2}+{F}_{x}^{2}}$$

### Statistical analysis

Statistical analyses were performed using generalized linear mixed models (GLMM) to compare the baselines as well as the progressions between patients and HI. We used the software R 3.6.0 (The R Foundation for Statistical Computing, Austria, Vienna, 2019) and the following packages: *nlme*, *ggplot2*, *dplyr*, and *tidyr*. The data for the biSAT variable were log-transformed to make the distribution of values more symmetric. The descriptive graphs used individual data that were calculated in 3-s intervals and smoothed with the loess algorithm by setting the bandwidth to 0.75 and using tricubic weighting.

Among the patients, the level of impairment of sensorimotor function as measured by the FMA-UE was quite variable (FMA-UE score range: 28 to 66). To take this heterogeneity into account, the patients were separated into two groups according to whether the FMA-UE score was equal to 66 (i.e., normal, Group 1, n = 9) or less than 66 (Group 2, n = 15) (Table 1).

At each stage (D1, D2, D3 and NC), the performances during the first and last minutes of training were calculated. The baseline was defined as the performance at the first 1-min block of D1, the overall progression was defined as the evolution between the beginning of D1 and the end of D3, and the generalization level was defined as the evolution between the baseline and the first 1-min block of the NC. For each variable, the baseline, overall progression and generalization levels were compared using a linear mixed model with group (HIs, 1 or 2) and time (in 8 levels: ‘D1 start’, ‘D1 end’, ‘D2 start’,’D2 end’,’D3 start’,’D3 end’, ‘NC start’, ‘NC end’) as fixed effects and a random intercept per individual as a random effect. Estimates of the effects within each group and differences between groups were accompanied by a 95% confidence interval (CI) and P-values as recommended in the CONSORT guidelines [42].

Correlations of performance measured on the CIRCUIT (baseline, overall progression and generalization on the NC) and baseline clinical scales (FMA-UE, SIS, MoCA, ABILHAND and BBT for the nonparetic hand) were assessed by Pearson's correlation coefficients, and they included all patients. For the interpretation of the results, we used the confidence interval (CI). The CI provides valuable insight into whether the trial result is compatible with a clinically important effect, regardless of the P value [42].

## Results

### Baseline characteristics of subjects

Between July 2019 and November 2019, 70 patients were screened for eligibility, with 24 meeting the inclusion criteria and agreeing to participate (see CONSORT flow diagram as Additional file 1: Fig. S2). No patient withdrawal or adverse events occurred during the experiment. The baseline characteristics of the subjects are given in Table 1.

### Evolution on the bimanual CIRCUIT task

**Fig. 2:**
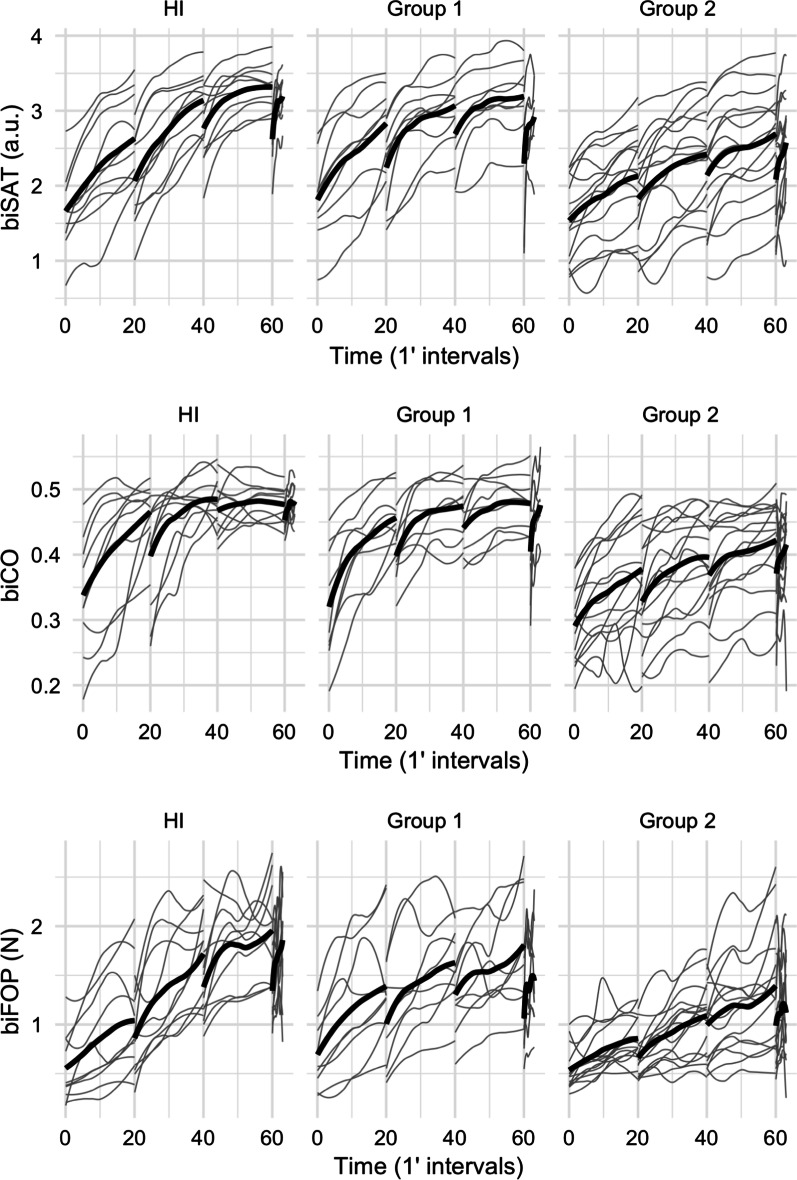
Improvement of biSAT, biCO, biFOP on the CIRCUIT task. biSAT, biCO (in arbitrary units, a.u.) and biFOP (in Newton) quantifying bimanual speed/accuracy trade-off (SAT), coordination between the velocities of the two hands and the bimanual forces exerted against the virtual walls, respectively. The thick lines correspond to the group means, the grey lines to individuals (HIs, patients from Group 1 and from Group 2, respectively). 0: baseline on D1; 0–20: training D1 n, 21–40: training D2; 41–60: training D3; 61–63: generalization (using a new CIRCUIT (NC) layout), HIs: healthy individuals (n = 10), Group 1: patients with stroke with FMA-UE = 66 (n = 9), Group 2: patients with stroke with FMA-UE < 66 (n = 15)

**Fig. 3:**
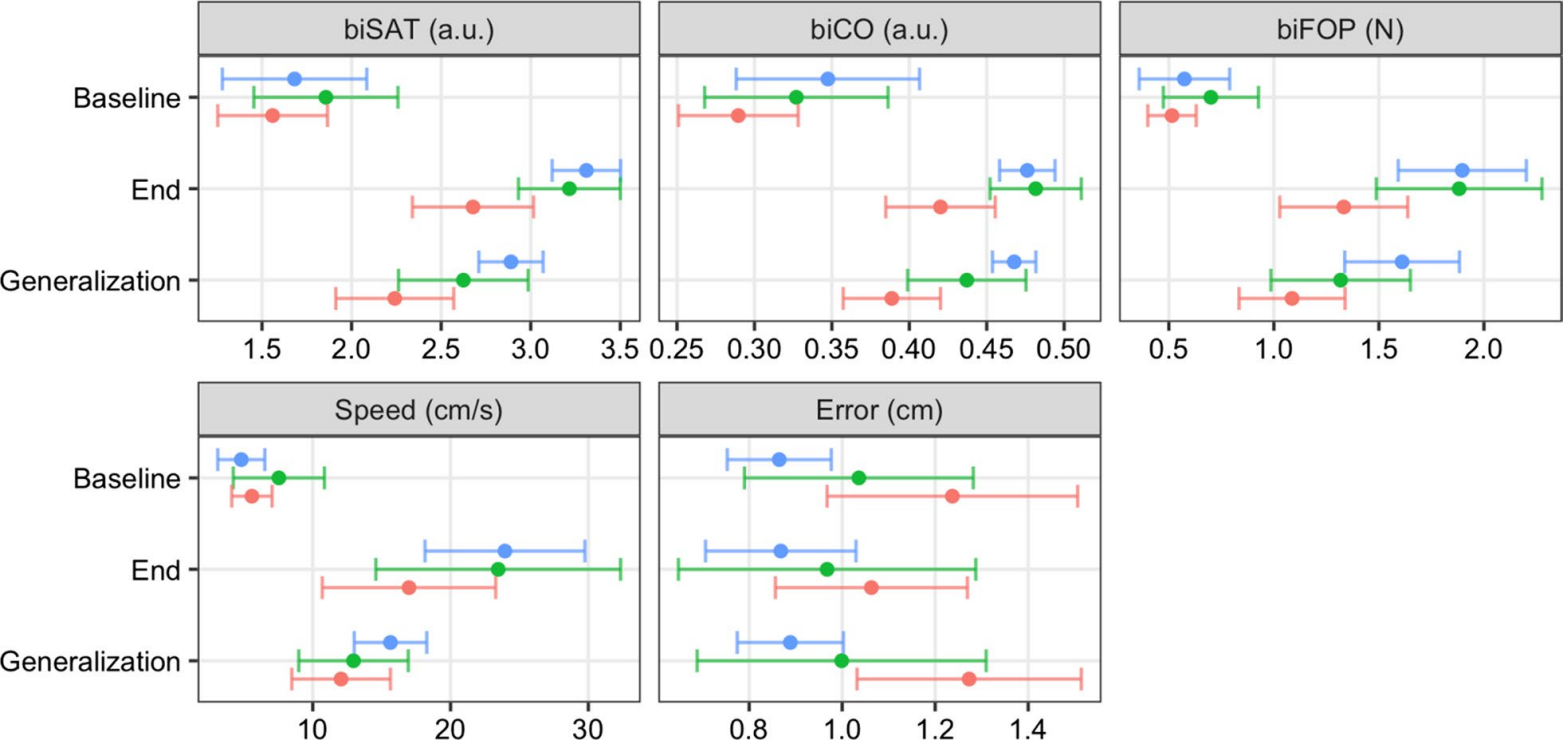
Results for the biSAT, biCO, biFOP, Speed and Error on a CIRCUIT task. Results are expressed as effect size (ES) and ± 95% confidence interval. The blue line corresponds to the healthy individuals (HIs) group, the green line to Group 1 (with FMA-UE = 66), and the red line to Group 2 (with FMA-UE < 66). The dots correspond to the effect sizes and the bars to the CI

In the HIs, a continuous progression of the biSAT was observed, with a slight loss overnight or when starting on the NC (i.e., at the beginning of the generalization test), a trend to plateau at the end of training on D3, and a large generalization to the new CIRCUIT (Figs. 2, 3, Additional file 1: Table S1 and Fig. S6). The HIs clearly achieved bim-MSkL.

The baseline biSAT was not significantly different between the HIs and patients from Groups 1 and 2 (Fig. 3, Table 2). In Group 2 (FMA-UE < 66), the progression of the biSAT followed a trend similar (general mean curve pattern) to that of the HIs while the biSAT improved significantly less overall compared with the HIs (− 0.5 [− 0.8; − 0.2], p = 0.0003) and the progression was slower (Figs. 2, 3, Table 2, Additional file 1: Fig. S6). Group 1 (FMA-UE = 66) showed an intermediate progression between the HIs and Group 2. In both the HIs and patients, the daily progression of the biSAT reflected an increase in velocity with a constant error, while the daily evolution of the biSAT (i.e., during training) was driven by an increase in velocity and a decrease in average error (Additional file 1: Fig. S3, S4, Table S1). When exposed to a new CIRCUIT after training on D3, the HIs achieved a larger biSAT generalization compared to the patients from Group 1 (− 0.4 [− 0.7; − 0.1] vs HIs, p = 0.017) and Group 2 (− 0.5 [− 0.8; − 0.3] vs HIs, p < 0.001)) (Figs. 2, 3, Table 2, Additional file 1: Fig. S6).

**Table 2:** Results of the CIRCUIT task

Phase	Variable	Group	Estimate	Lower	Upper	P-value
Baseline	biSAT	1 vs HIs	0.18	− 0.36	0.71	0.5104
Baseline	biSAT	2 vs HIs	− 0.12	− 0.6	0.35	0.604
Baseline	biSAT	1 vs 2	− 0.3	− 0.79	0.19	0.2269
Overall	biSAT	1 vs HIs	− 0.27	− 0.58	0.04	0.0862
Overall	biSAT	2 vs HIs	− 0.51	− 0.78	− 0.24	**0.0003**
Overall	biSAT	1 vs 2	− 0.24	− 0.52	0.04	0.0951
Generalization	biSAT	1 vs HIs	− 0.38	− 0.7	− 0.07	**0.0177**
Generalization	biSAT	2 vs HIs	− 0.53	− 0.8	− 0.25	**0.0002**
Generalization	biSAT	1 vs 2	− 0.15	− 0.43	0.14	0.3234
Baseline	biCO	1 vs HIs	− 0.02	− 0.08	0.04	0.4793
Baseline	biCO	2 vs HIs	− 0.06	− 0.11	− 0.01	**0.0291**
Baseline	biCO	1 vs 2	− 0.04	− 0.09	0.02	0.1612
Overall	biCO	1 vs HIs	0.03	− 0.02	0.08	0.3054
Overall	biCO	2 vs HIs	0	− 0.04	0.05	0.9355
Overall	biCO	1 vs 2	− 0.02	− 0.07	0.02	0.299
Generalization	biCO	1 vs HIs	− 0.01	− 0.06	0.04	0.7185
Generalization	biCO	2 vs HIs	− 0.02	− 0.07	0.02	0.3435
Generalization	biCO	1 vs 2	− 0.01	− 0.06	0.03	0.6125
Baseline	biFOP	1 vs HIs	0.13	− 0.28	0.54	0.5344
Baseline	biFOP	2 vs HIs	− 0.06	− 0.42	0.3	0.7426
Baseline	biFOP	1 vs 2	− 0.19	− 0.56	0.19	0.3224
Overall	biFOP	1 vs HIs	− 0.14	− 0.47	0.19	0.4015
Overall	biFOP	2 vs HIs	− 0.51	− 0.8	− 0.21	**0.0008**
Overall	biFOP	1 vs 2	− 0.36	− 0.67	− 0.06	**0.0187**
Generalization	biFOP	1 vs HIs	− 0.4	− 0.74	− 0.06	**0.0202**
Generalization	biFOP	2 vs HIs	− 0.46	− 0.76	− 0.17	**0.002**
Generalization	biFOP	1 vs 2	− 0.07	− 0.38	0.24	0.6783

Results are expressed as effect size (ES) and ± 95% confidence interval (CI). Estimate: estimates of effects within each group, Lower: lower limit for the mean, Upper: upper limit for the mean, biSAT: Bimanual Speed/Accuracy Trade-off in arbitrary units (a.u.), biCO: Bimanual Coordination Factor (a.u.), biFOP: bimanual forces exerted against the virtual walls (in Newtons), HIs: healthy individuals, 1: Group 1 (i.e., patients with FMA-UE = 66), 2: Group 2 (i.e., patients with FMA-UE < 66), Baseline: first CIRCUIT training block on the first day, Overall: overall progression over the three days consecutives, Generalization: task on a new CIRCUIT (NC) layout

In both HIs and patients, the biCO broadly followed the same trend as the biSAT, although Group 2 had poorer baseline bimanual coordination than the HIs (− 0.06 [− 0.11; − 0.01], p = 0.03) (Figs. 2, 3, Table 2, Additional file 1: Fig. S6). Interestingly, the biCO appeared to plateau at D3 in the HIs and Group 1, which was inconsistent with that of Group 2 (Fig. 2, Additional file 1: Fig. S6). However, the overall biCO progression was not significantly different among the three groups and its generalization on a new CIRCUIT did not significantly differ.

In the His and Groups 1 and 2, the biFOP also broadly followed the same trend as the biSAT (Figs. 2, 3, Additional file 1: Fig. S6). The baseline biFOP was not significantly different between groups. The overall biFOP progression was significantly smaller in Group 2 than the HIs (− 0.51 [− 0.8; − 0.21], p < 0.001) and Group 1 (− 0.36 [− 0.67; − 0.06], p = 0.018). Generalization for the biFOP was larger in the HIs than in Group 1 (− 0.4 [− 0.74; − 0.06] vs HIs, p = 0.02) and Group 2 (− 0.46 [− 0.76; − 0.17] vs HIs, p = 0.002).

### Generalization on the REACHING task and the BBT

From D1 to D3, the HIs achieved a clear generalization on the bimanual REACHING task (Table 3). An improvement trend was observed for the BBT for both the dominant and nondominant UL, whereas the GF remained stable (Table 3).

**Table 3:** Hand dexterity and grip force

Task of generalization	Variable	Hand	Day	HIs	Group 1	Group 2
				Means ± SD	Means ± SD	Means ± SD
REACHING	biSAT(a.u.)	Bimanual	D1	2.7 ± 0.8	2.5 ± 0.6	1.9 ± 0.6
			D3	4.0 ± 0.5	2.8 ± 0.6	2.7 ± 0.6
	biCO (a.u.)	Bimanual	D1	0.28 ± 0.07	0.30 ± 0.05	0.27 ± 0.04
			D3	0.38 ± 0.03	0.32 ± 0.03	0.38 ± 0.05
	biFOP (N)	Bimanual	D1	0.4 ± 0.3	0.5 ± 0.3	0.3 ± 0.1
			D3	1.1 ± 0.3	0.7 ± 0.4	0.7 ± 0.2
CLINICAL SCALES	BBT	Non-paretic / Dominant	D1	70 ± 10	65 ± 6	58 ± 9
			D3	75 ± 10	68 ± 6	62 ± 10
		Paretic / Non-dominant	D1	68 ± 10	59 ± 10	24 ± 18
			D3	74 ± 11	62 ± 9	25 ± 19
	GF	Non-paretic / Dominant	D1	34 ± 12	30 ± 11	33 ± 9
			D3	34 ± 12	29 ± 9	35 ± 10
		Paretic/ Non-dominant	D1	33 ± 10	28 ± 10	18 ± 7
			D3	33 ± 10	28 ± 8	18 ± 7

*BBT* Box and Block Test, *GF* grip force with Jamar Hydraulic Hand Dynamometer®, D1: Day 1, D3: Day 3

The patients from Groups 1 and 2 also showed generalization from the CIRCUIT to bimanual REACHING tasks (Table 3), and a significant difference was observed between the two groups of subjects. Similarly, an improvement trend was observed for the BBT for the paretic and nonparetic UL for Group 1. Group 2 showed an improvement only for the nonparetic UL, whereas the GF remained stable, which was also observed in the HIs.

### Correlation analyses in patients

The correlations between bim-MSkL outcomes and baseline clinical scales are displayed in Additional file 1: Fig. S5. The FMA-UE was positively correlated with the overall progression of the biSAT (r = 0.36 [0.03; 0.62]) and biFOP (r = 0.42 [0.09; 0.66]). The SIS was correlated with the overall progression of the biSAT (r = 0.44 [0.03; 0.73]) and with the generalization of the biSAT (r = 0.44 [0.00; 0.73]) and biCO (r = 0.45 [0.02; 0.74]). The BBT of the paretic hand was correlated with the overall progression of the biSAT (r = 0.42 [0.09; 0.67]) and biFOP (r = 0.46 [0.14; 0.69]) as well as with the generalization on biSAT (r = 0.44 [0.11; 0.68]). The MoCA was correlated with the baseline biSAT (r = 0.5 [0.12; 0.75]). Finally, the ABILHAND did not correlate with the robotic outcomes.

## Discussion

When training with a serious game on a neurorehabilitation robot, patients in the chronic phase of stroke were able to learn and retain a complex bimanual skill and to generalize performance improvements to other bimanual or unimanual tasks. The HIs performed better than the patients with more severe impairment (Group 2, FMA-UE: 28–65), who showed large interindividual variability in both the magnitude and trajectory of bim-MSkL. The patients with minimal impairment (Group 1, FMA-UE: 66) showed intermediate progression between the HIs and Group 2.

### Bimanual motor skill learning

Across sessions, the HIs showed changes in the biSAT, biCO and biFOP as well as retention and generalization, and they achieved typical bim-MSkL. Overall, chronic patients with supratentorial stroke were able to achieve complex bim-MSkL involving a new control policy and to generalize performance improvements to a new, untrained, complex bimanual task. The first hypothesis of this study was thus confirmed. These data expand the results from a previous study in which patients in the chronic phase of stroke achieved bim-MSkL over a single training session under real and sham tDCS, and an additional effect of noninvasive brain stimulation was not observed [29]. In the current study, changes in the three outcomes (biSAT, biCO, and biFOP) were observed across three consecutive days. Compared to the last block on the previous day, a slight performance drop was observed for the first block of D2 and D3, although overnight retention remained consistent.

Given that 9 patients in the chronic phase of stroke had a normal FMA-UE score, we decided to split the patient pool into two groups. In Group 1 (FMA-UE = 66, n = 9), the overall progression of the biSAT was not significantly different compared to that of the HIs. In Group 2 (FMA-UE < 66, n = 15), the overall progression of the biSAT was significantly inferior to that of the HIs. Nevertheless, patients from Group 2 achieved bim-MSkL, including retention and generalization, and did not seem to reach a ceiling; however, whether their ability could eventually match that of Group 1 after further training was not determined.

The overall biCO progression was not significantly different between the three groups, although Group 2 had poorer baseline bimanual coordination than the HIs. Interestingly, the biCO appeared to plateau at D3 in the HIs and Group 1, whereas this was not observed in Group 2. In a previous study in younger HIs, the biCO was correlated with the biSAT [29], suggesting that the biSAT and biCO reflect either the same process or overlapping processes. It is possible that within their range of potential biCO improvement, the patients (Groups 1 and 2) did not perform significantly worse than the His; however, this did not translate into similar improvements on the primary outcome (biSAT), for which feedback was provided.

In the current study, the biFOP increased across sessions in both patients and HIs, which was inconsistent with the decrease observed previously in younger HIs [41]. The biFOP quantifies the forces exerted in nondesired directions by each hand against virtual walls. Theoretically, training should result in improvement, and the biFOP should thus *decrease*, which would reflect less force “wasted” in the wrong direction [41]. Here, instruction about the force was not provided and a penalty was not assigned for pushing against the virtual walls. Therefore, the HIs and patients might have simply not paid attention to this aspect and remained focused on the biSAT (for which feedback was provided) at the cost of some increase in the biFOP.

To summarize, the HIs and patients from Groups 1 and 2 achieved bim-MSkL over three days, including overnight retention. The progression of Group 1 was intermediate between that of the HIs and Group 2, in which robotic outcome improvements remained globally inferior to that of the HIs, suggesting that there was still room for improvement in more impaired patients.

### Generalization

The HIs achieved a larger biSAT generalization and biFOP increase compared to the patients from Groups 1 and 2, whereas the biCO generalization was similar across the groups. Thus, both the HIs and patients were able to generalize the newly learned bimanual control policy, which is a hallmark of MSkL [43].

Furthermore, the improvements driven by the bimanual CIRCUIT training transferred to bimanual REACHING. The HIs and patients were thus able to use the newly learned bimanual control policy to achieve a different task within the same robotic environment. Finally, in both the HIs and patients, there was also a trend for a (transfer of) performance improvement to the unimanual BBT, whereas the unimanual GF remained unchanged. Of course, we cannot rule out that this improved trend is actually due to the short interval between test and retest. Although the BBT improvements remained modest in patients (see Table 3), this finding is encouraging for neurorehabilitation but it remains to be confirmed in future experiments.

### Correlations between robotic outcomes and clinical scales

The FMA-UE did not correlate with the baseline biSAT, biCO or biFOP, suggesting that the degree of unilateral motor impairment was not an accurate predictor of how well chronic patients could coordinate bimanual movements. Interestingly, the biSAT evolution correlated positively with the FMA-UE, BBT and SIS, suggesting that patients with less baseline impairment and participation restriction could achieve larger bim-MSkL after training on a robotic device. Although the lack of correlation between the bimanual robotic outcomes and the ABILHAND is surprising at first glance, it may reflect a discrepancy between the bimanual ADL the patients believe they are able to achieve and the tasks that can be objectively quantified with a bimanual robotic system. Furthermore, the ABILHAND questionnaire does not consider compensation while performing these ADL, whereas compensation is limited during evaluations with the REAplan®.

### Therapeutic implications

Our data demonstrate that bim-MSkL with the REAplan® robot may help improve bimanual coordination in patients with chronic stroke with either minimal or mild-moderate impairment, which may indicate interesting prospects for neurorehabilitation [44]. Previous rehabilitation studies have shown improvements in bilateral limb functions after stroke, such as bilateral arm training with rhythmic auditory cueing (BATRAC) and robotic mirror image movement enabler (MIME) [45]. It might be interesting to perform more randomized control trials that implement cooperation between the ULs, such as training to perform asymmetrical bimanual actions that sharing a common goal, and to compare bimanual with unimanual interventions [44, 46].

In this study, we used an active mode (i.e., no robotic assistance), which requires a minimal level of residual function of the paretic UL to perform the bimanual tasks. It would be interesting to investigate other RAT modes, e.g., passive and/or active-assisted mode with an adaptive algorithm [31], in patients with more severe motor or cognitive impairments.

### Limitations

This study has several limitations. The sample size was relatively small and heterogeneous (e.g., strokes with different sizes, residual impairments, time since stroke, etc.). Furthermore, the patients had mostly mild to moderate UL impairment (FMA-UE: 56.6 ± 13.5, range 28–66). It is unknown whether similar results would be found in patients with more severe impairments. Moreover, on D3, the duration of the robotic session was longer than on the previous days. Indeed, the patients performed the Generalization task (three 1-min runs) and the REACHING task (16 trials, back and forth) in addition to the training. It is possible that this led to a decrease in performance at the end of on D3, due to either physical or cognitive fatigue. Large interindividual variability was observed, and it would be interesting to correlate bim-MSkL with the size and localization of the stroke on brain imaging. One method of confirming and expanding these results would be to recruit more patients. Next, the improvement in bim-MSkL was large over three consecutive days of training and the biCO seemed to plateau in Group 1 on D3, such as in the HIs. It is unknown whether expanding the number of training sessions would further strengthen the improvements. However, combining several unimanual and bimanual serious games on a robotic device might enhance recovery further. Finally, it would be interesting to combine bimanual RAT with “classical” neurorehabilitation.

## Conclusions

The HIs and chronic patients with supratentorial stroke with either minimal or mild-moderate impairment were able to improve and retain a complex bimanual skill over three consecutive days. Furthermore, they could generalize their performance improvements to different tasks. These results demonstrate that bim-MSkL can be achieved in patients with chronic stroke by means of a robotic interface that displays a serious game, and these findings indicate that the proposed method has interesting possibilities for neurorehabilitation.

## Supplementary Information

**Additional file 1: Fig. S1.** Formula of the biCO for the REACHING task. **Fig. S2.** Simplified CONSORT flow diagram for the chronic stroke patients. **Table S1.** Speed and Error on the CIRCUIT task. **Fig. S3.** Evolution of velocity on the CIRCUIT task. **Fig. S4.** Evolution of error on the CIRCUIT task. **Fig. S5.** Correlations between robotic outcomes and baseline clinical scales. **Fig. S6.** Overlap of the progressions of the groups.

## Data Availability

The datasets for this study are available from the corresponding author on reasonable request.

## Abbreviations

ADL: Activities of daily life
biCO: Bimanual coordination factor
biFOP: Bimanual forces
Bim-MSkL: Bimanual motor skill learning
biSAT: Bimanual speed/accuracy trade-off
HIs: Healthy individuals
ULs: Upper limbs
RAT: Robot-assisted training
