# Supplementary material for: Bimanual motor skill learning with robotics in chronic stroke: comparison between minimally impaired and moderately impaired patients, and healthy individuals
Source: J Neuroeng Rehabil. 2022 Mar 17;19:28. doi: 10.1186/s12984-022-01009-3 (PMC8928664; doi:10.1186/s12984-022-01009-3)
Supplement: Supplementary file 1 — Additional file 1: Fig. S1. Formula of the biCO for the REACHING task. Fig. S2. Simplified CONSORT flow diagram for the chronic stroke patients. Table S1. Speed and Error on the CIRCUIT task. Fig. S3. Evolution of velocity on the CIRCUIT task. Fig. S4. Evolution of error on the CIRCUIT task. Fig. S5. Correlations between robotic outcomes and baseline clinical scales. Fig. S6. Overlap of the progressions of the groups. [file 12984_2022_1009_MOESM1_ESM.docx]

**Additional Materials**

**Additional file 1. General functioning of the REAplan®.**

With the distal effector of the REAplan® (AXINESIS, Wavre, Belgium) robot, the subjects are able to realize displacements in the horizontal plane through movement of the shoulder (flexion/extension and /adduction/abduction) and the elbow (flexion/extension), by interacting with the robot through a handle mounted on a forearm rest gutter. The height of the table (i.e., the horizontal plane), is adjusted to the subject’s morphology in order to provide a comfortable sitting position.

A large immersive screen is positioned in front of the subject, on which the instructions, tasks and feedbacks are displayed. By using a connected keyboard and a side-monitor, the therapist can configure the exercises, start and stop trials, and monitor the progression of the subject’s performances.

The distal effectors (handles and forearms gutters) are positioned in front of each shoulder of the subject during the calibration phase. For the current experiment, depending on the randomization, each UL controlled the cursor’s displacement along a single axis, either left-right or front-back (i.e., lateral X-axis or sagittal Y-axis).

**Fig. S1. Formula of the biCO for the REACHING task.**

$$biCO=\frac{min(\left| V_{x} \right|,\left| V_{y} \right|*0.41)}{\sqrt{\left( V_{x} \right)^{2}+(0.41*V_{y})^{2}}}$$

Because in the ± 22.5° targets (from the Y-axis), the ideal coordination does not happen when V_x_ = V_y_ but when V_x_ is 0.41 x V_y_ (i.e. tan 22.5° = V_x_/V_y_ = 0.41). Finally, as 0° REACHING was actually “unimanual”, biCO was not calculated.

**Fig. S2. Simplified CONSORT flow diagram for the chronic stroke patients.**

## Enroll

## ment

Assessed for eligibility (n=70)

Analysed (n=11)
♦ Excluded from analysis (give reasons) (n=0)

Analysed (n=13)
♦ Excluded from analysis (give reasons) (n=0)

Lost to follow-up (give reasons) (n= 0)

Discontinued intervention (give reasons) (n= 0)

Lost to follow-up (give reasons) (n=0)

Discontinued intervention (give reasons) (n= 0)

Allocated to intervention: Left-UpDown. (n=13)

♦ Received allocated intervention (n=13)

♦ Did not receive allocated intervention (give reasons) (n=0)

Allocated to intervention Right-UpDown (n= 11)

♦ Received allocated intervention (n=11)

♦ Did not receive allocated intervention (give reasons) (n= 0)

## Allocation

## Follow-Up

Excluded (n= 46)

♦  Not meeting inclusion criteria (n=7)

♦  Declined to participate (n= 2)

♦  Other reasons (n= 37)

- Others diseases (n= 8)
- Not disponible 3 days (n= 17)
- Do not wish (n=12)

Randomized (n= 24)

## Analysis

**Table S1. Speed and Error on the CIRCUIT task.**

|  | **Speed (cm/s)**  Mean ± SD | | |  | **Error (cm)**  Mean ± SD | | |
| --- | --- | --- | --- | --- | --- | --- | --- |
|  | **HIs** | **Group 1** | **Group 2** |  | **HIs** | **Group 1** | **Group 2** |
| D1 start | 5 ± 3 | 8 ± 5 | 6 ± 3 |  | 0.86 ± 0.18 | 1.04 ± 0.37 | 1.24 ± 0.52 |
| D1 end | 10 ± 7 | 16 ± 9 | 9 ± 4 |  | 0.66 ± 0.11 | 0.97 ± 0.54 | 1.16 ± 0.51 |
| D2 start | 7 ± 4 | 10 ± 6 | 7 ± 4 |  | 0.94 ± 0.27 | 1.11 ± 0.47 | 1.22 ± 0.54 |
| D2 end | 18 ± 7 | 19 ± 8 | 12 ± 6 |  | 0.81 ± 0.28 | 0.94 ± 0.48 | 1.11 ± 0.50 |
| D3 start | 15 ± 5 | 14 ± 7 | 11 ± 6 |  | 0.91 ± 0.30 | 1.07 ± 0.53 | 1.28 ± 0.54 |
| D3 end | 24 ± 9 | 23 ± 13 | 17 ± 12 |  | 0.87 ± 0.26 | 0.97 ± 0.48 | 1.06 ± 0.40 |
| NC start | 16 ± 4 | 13 ± 6 | 12 ± 7 |  | 0.89 ± 0.18 | 1.00 ± 0.44 | 1.27 ± 0.47 |
| NC end | 20 ± 7 | 17 ± 9 | 14 ± 9 |  | 0.89 ± 0.28 | 1.04 ± 0.54 | 1.13 ± 0.41 |
| R1 | 17 ± 12 | 15 ± 11 | 9 ± 5 |  | 0.90 ± 0.19 | 1.03 ± 0.45 | 1.32 ± 0.72 |
| R2 | 49 ± 18 | 14 ± 9 | 18 ± 12 |  | 0.85 ± 0.17 | 0.77 ± 0.31 | 1.08 ± 0.45 |

**Table S1.** D1: day 1, D2: day 2, D3: day 3, NC: New Circuit layout for assessed the generalization, R1: reaching on D1, R2: reaching on D2), HIs: healthy ndividuals, 1: Group 1 (i.e., patients with FMA-UE =66), 2: Group 2 (i.e., patients with FMA-UE <66).

**Fig. S3. Evolution of velocity on the CIRCUIT task.**

**
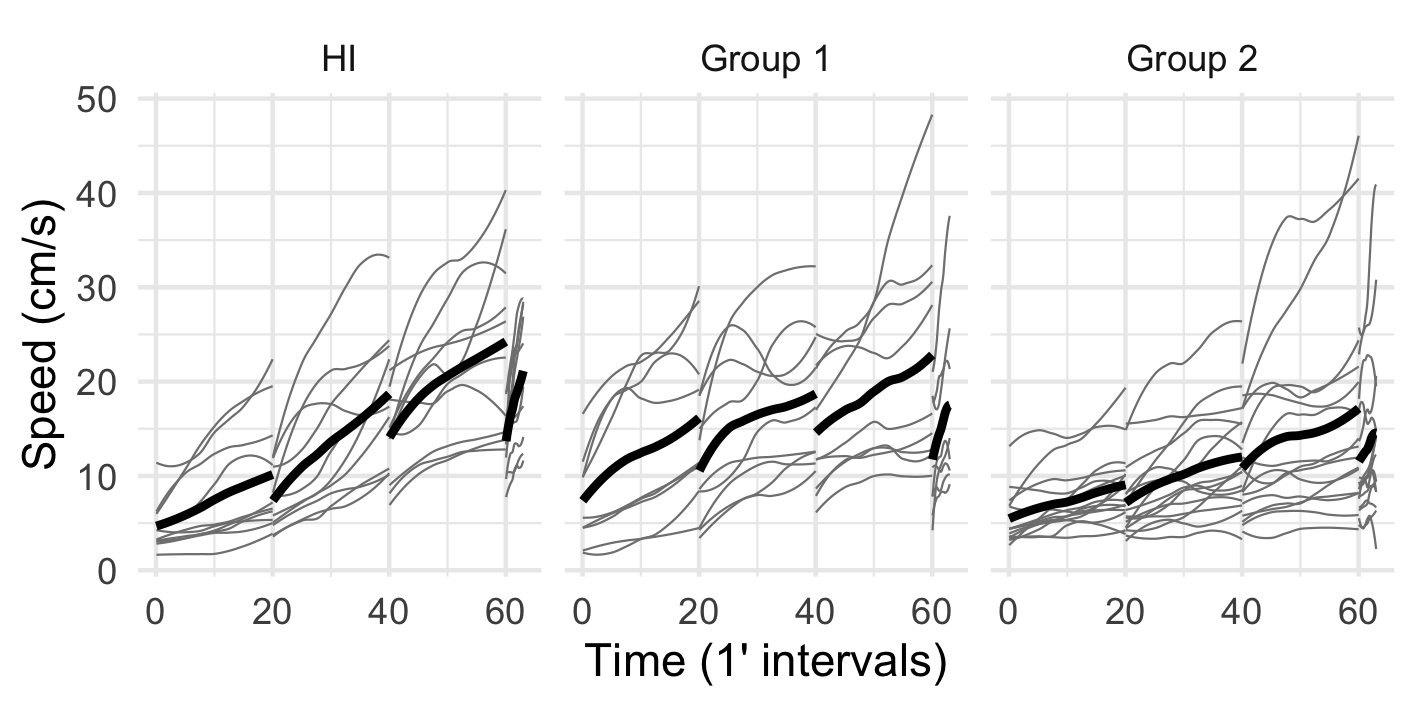
**

Velocity measured in cm/s over the three days of training on the CIRCUIT task. The thick black lines correspond to the group means, and the thin gray lines correspond to the healthy individuals (HIs), patients with stroke with FMA-UE = 66 (Group 1), and patients with stroke with FMA-UE < 66 (Group 2) respectively. D1: blocks 1-20, D2: blocks 21-40, D3: blocks 41-60, NC (generalisation on D3): blocks 61-63.

**Fig. S4. Evolution of error on the CIRCUIT task.**

**
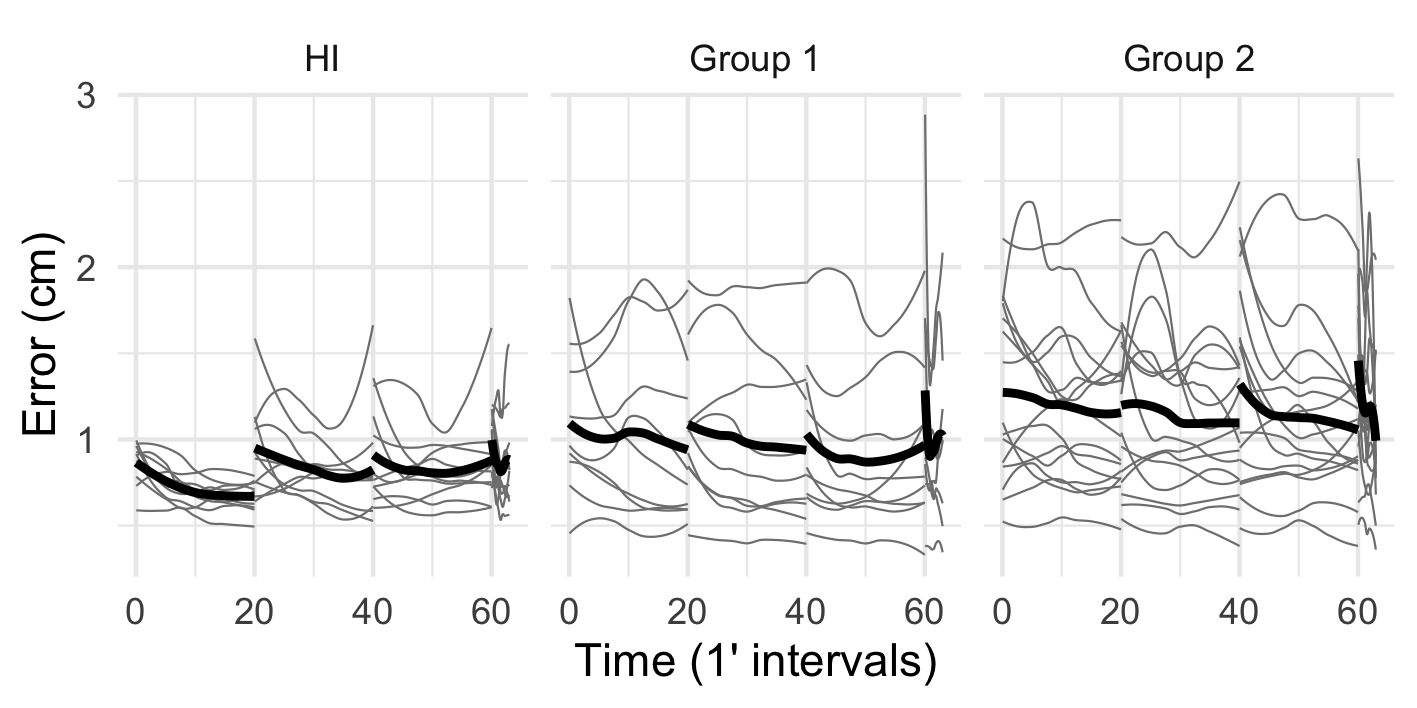
**

Error (distance between the cursor trajectory and the ideal path, defined as the centre of the track, in cm. Same conventions as for Supplementary Figure 3.

**Fig. S5. Correlations between robotic outcomes and baseline clinical scales.**


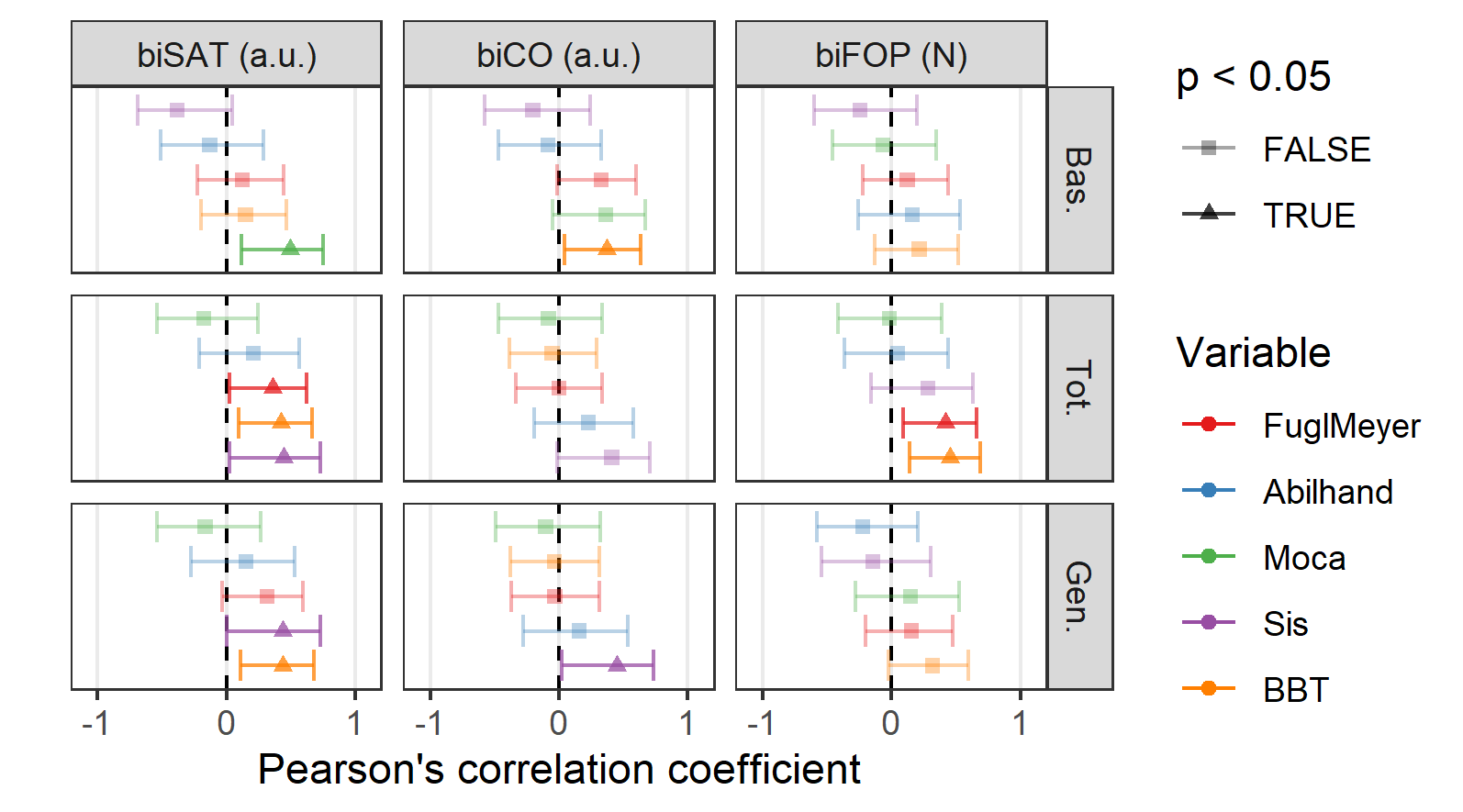


**Fig. S5.** Pearson’s correlation coefficient for each pair of

variables of the 95% confidence interval (CI). biSAT/biCO/biFOP: variables

representing the progressions on the robot, Bas: baseline (i.e., first CIRCUIT training block on the first day, Tot: overall progression, Gen: generalization (i.e., task on a new CIRCUIT (NC) layout), Fugl-Meyer Upper Extremity (FMA-UE), ABILHAND: measure of manual ability for patients with chronic stroke in activities of daily living, MoCA: Montreal Cognitive Assessment, Stroke Impact Scale (SIS) and Box and Blocks Test (BBT). The dots correspond to the effect sizes and the bars to the 95% CI. The dashed line represents the value 0 (no correlation), the (0, 1) interval correspond to a positive correlation, the (-1, 0) interval correspond to a negative correlation. Here for the interpretation of the results we used the confidence interval (CI). The CI provides valuable insight into whether the trial result is compatible with a clinically important effect, regardless of the P-value (39). TRUE correspond to a significant correlation (i.e., the P-value < 0.05). FALSE correspond to non-significant correlation (i.e., P-value >= 0.05).

**Fig. S6. Overlap of the progressions of the groups.**


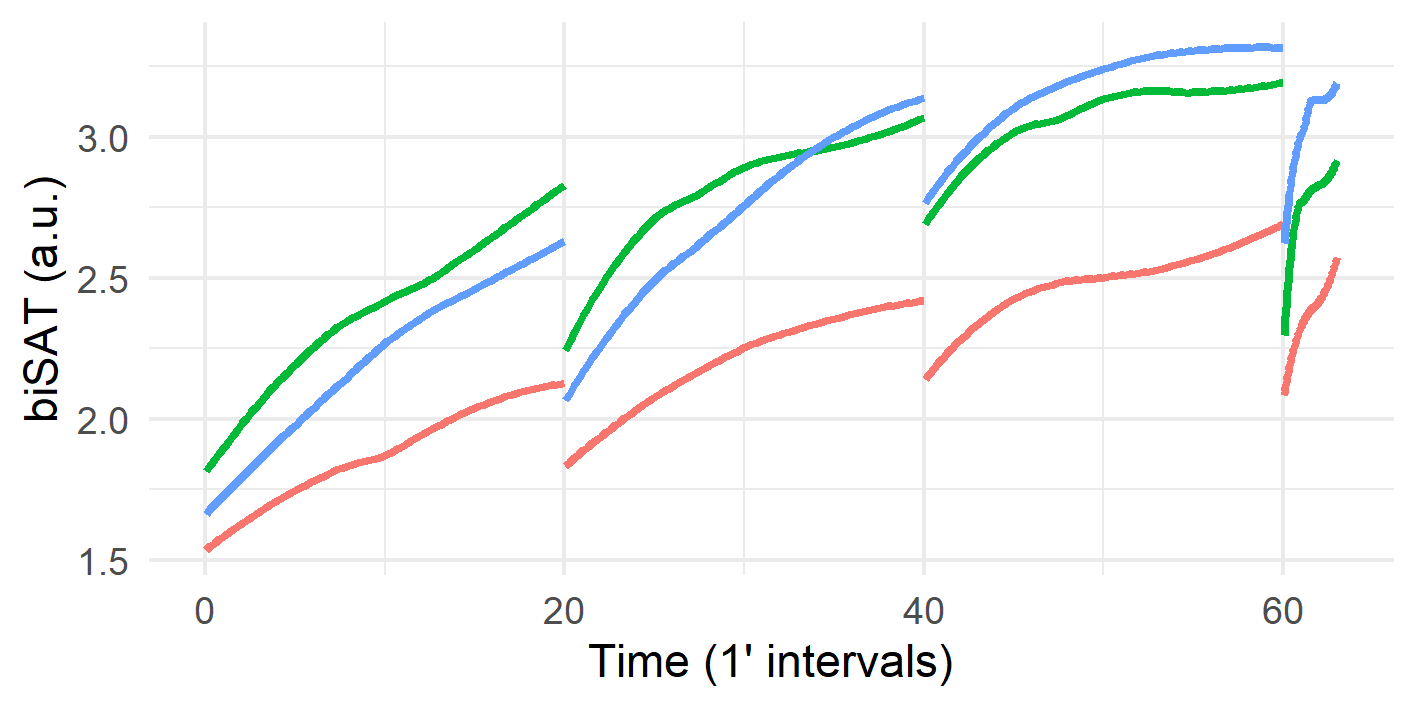

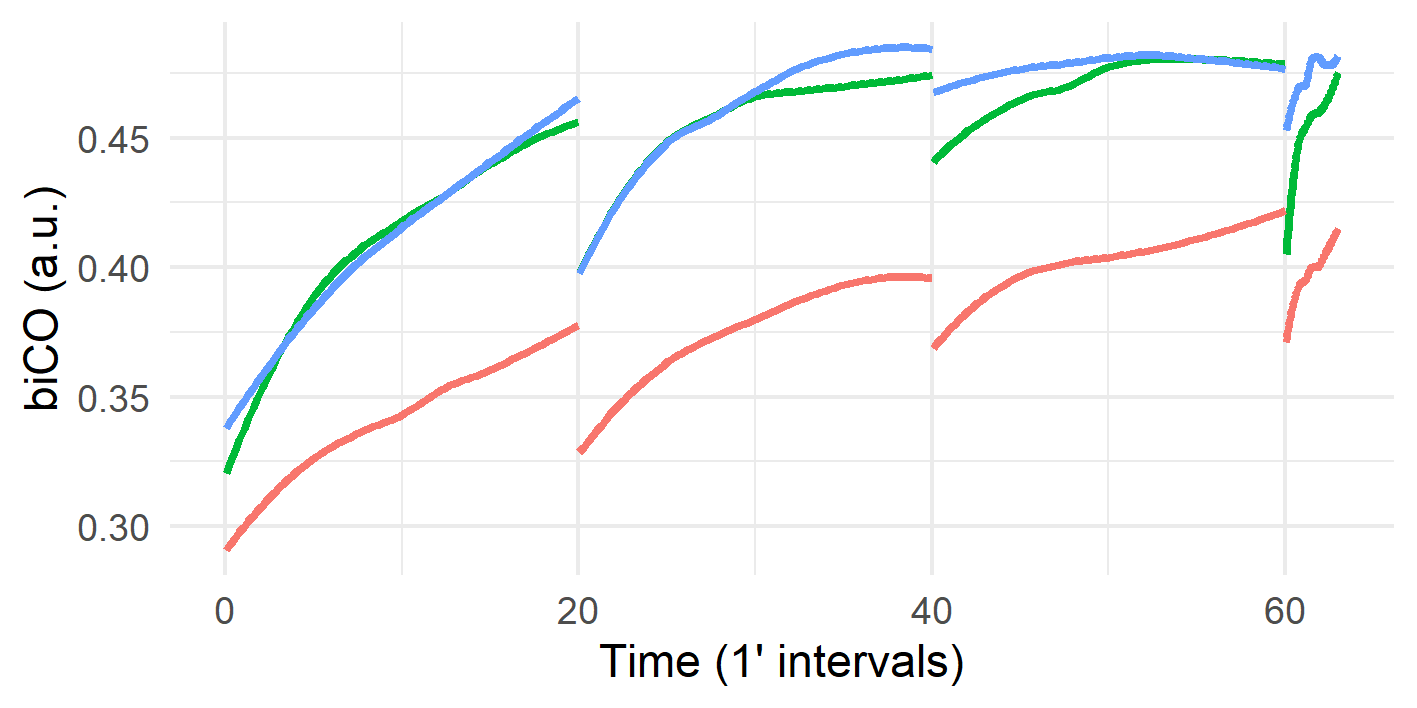

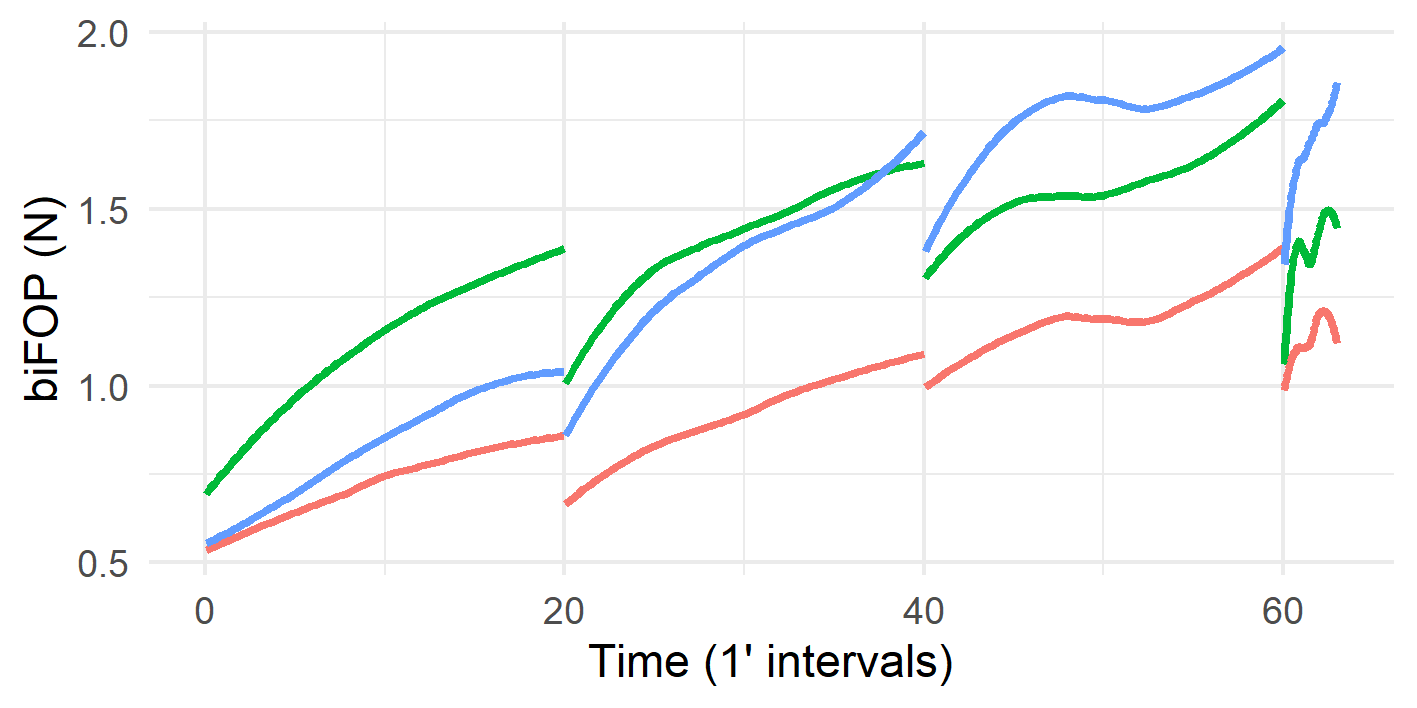


**Fig. S6.** biSAT, biCO (in arbitrary units, a.u.) and biFOP (in Newton) quantifying bimanual speed/accuracy trade-off (SAT), coordination between the velocities of the two hands and the bimanual forces exerted against the virtual walls, respectively. The thick lines correspond to the group means: the blue line corresponds to the healthy individuals (HIs) group, the green line to stroke Group 1 (with FMA-UE = 66), and the red line to Group 2 (with FMA-UE < 66). 0: baseline on D1; 0-20: training D1 (20 blocks of 1-min training blocks), 21-40: training D2; 41-60: training D3; 61-63: generalization (using a new CIRCUIT (NC) layout).
